# Avoidable mortality due to long-term exposure to PM_2.5_ in Colombia 2014–2019

**DOI:** 10.1186/s12940-022-00947-8

**Published:** 2022-12-24

**Authors:** Laura A. Rodriguez-Villamizar, Luis Carlos Belalcazar-Ceron, María Paula Castillo, Edwin Ricardo Sanchez, Víctor Herrera, Dayana Milena Agudelo-Castañeda

**Affiliations:** 1 Department of Public Health, Universidad Industrial de Santander, Carrera 32 29-31 Of. 301 Facultad de Salud, 68002 Bucaramanga, Colombia; 2 School of Engineering, Universidad Nacional de Colombia, Bogotá, Colombia; 3 Faculty of Health Sciences, Universidad Autónoma de Bucaramanga, Bucaramanga, Colombia; 4 Department of Civil and Environmental Engineering, Universidad del Norte, Barranquilla, Colombia

**Keywords:** Air pollution, Particulate matter, Reference standards, Satellite imagery, Mortality, Colombia

## Abstract

**Objective:**

To compare estimates of spatiotemporal variations of surface PM_2.5_ concentrations in Colombia from 2014 to 2019 derived from two global air quality models, as well as to quantify the avoidable deaths attributable to the long-term exposure to concentrations above the current and projected Colombian standard for PM_2.5_ annual mean at municipality level.

**Methods:**

We retrieved PM_2.5_ concentrations at the surface level from the ACAG and CAMSRA global air quality models for all 1,122 municipalities, and compare 28 of them with available concentrations from monitor stations. Annual mortality data 2014–2019 by municipality of residence and pooled effect measures for total, natural and specific causes of mortality were used to calculate the number of annual avoidable deaths and years of potential life lost (YPLL) related to the excess of PM_2.5_ concentration over the current mean annual national standard of 25 µg/m^3^ and projected standard of 15 µg/m^3^.

**Results:**

Compared to surface data from 28 municipalities with monitoring stations in 2019, ACAG and CAMSRA models under or overestimated annual mean PM_2.5_ concentrations. Estimations from ACAG model had a mean bias 1,7 µg/m^3^ compared to a mean bias of 4,7 µg/m^3^ from CAMSRA model. Using ACAG model, estimations of total nationally attributable deaths to PM_2.5_ exposure over 25 and 15 µg/m^3^ were 142 and 34,341, respectively. Cardiopulmonary diseases accounted for most of the attributable deaths due to PM_2.5_ excess of exposure (38%). Estimates of YPLL due to all-cause mortality for exceeding the national standard of 25 µg/m^3^ were 2,381 years.

**Conclusion:**

Comparison of two global air quality models for estimating surface PM_2.5_ concentrations during 2014–2019 at municipality scale in Colombia showed important differences. Avoidable deaths estimations represent the total number of deaths that could be avoided if the current and projected national standard for PM_2.5_ annual mean have been met, and show the health-benefit of the implementation of more restrictive air quality standards.

**Supplementary Information:**

The online version contains supplementary material available at 10.1186/s12940-022-00947-8.

## Introduction

Exposure to air pollutants have adverse effects on human health leading to increased mortality. Although various atmospheric pollutants are associated with increased risk of mortality, especially for respiratory and cardiovascular diseases, atmospheric particulate matter < 2.5 μm -PM_2.5_- is widely studied and is often used as a proxy indicator of air pollution exposure [1]. PM_2.5_ consists of inhalable particles and its adverse effects are due to their capacity to penetrate and deposit into the lower respiratory tract, facilitating the submicron particles to avoid the tissues’ natural mechanisms of clearance and to form active oxides into the lungs [2, 3]. These are the most cytotoxic ambient particles and as a result, there is a long- term retention of the particles and their absorbed chemicals cause oxidative damage and an increase in the risk of toxicity [2, 4–6].

There is vast epidemiological evidence of the association between PM_2.5_ and mortality and morbidity outcomes [7–10]. The International Agency of Research on Cancer (IARC) have raised environmental concerns about atmospheric particles affecting air quality and human health and declared PM in outdoor pollution as carcinogenic to humans [11]. In 2017, it was estimated that 92% of the world’s population lived in areas that exceeded the World Health Organization (WHO) Air Quality Guidelines (AQG) 2005 for PM_2.5,_ thus contributing to 2.9 million deaths [12]. The burden of disease caused by ambient air pollution is large, particularly in low- and middle-income countries, being the leading environmental risk factor and one of the most important overall risk factors for global mortality [13, 14]. It is estimated that ambient air pollution is responsible for 4 to 9 million deaths each year worldwide, and therefore reducing air pollutants concentrations has become a global goal related to achieving the Sustainable Development Goals (SDGs) by making more restrictive air quality regulations [15].

In Colombia, according to the national burden of environmental disease study, there were 15,361 deaths attributable to air pollution in 2016 (rate 719.18 per 100,000 population) [16]. Increases in PM_10_ concentrations have been associated with increased risk of cardiovascular mortality in Bogotá [17], and increases in PM_2.5_ levels have been associated with increased risk of cardiopulmonary morbidity in different Colombian cities [18]. Particulate matter, both PM_2.5_ and PM_10_, are the air pollutants of most concern by environmental authorities in Colombia as they are the pollutants with more exceedances per year based on national regulatory levels [19].

The evolution of air quality regulations has been dynamic, and air quality regulations have been created and updated. Colombia has had exclusive regulations for the control of air pollution. In 2010, the Air Pollution Prevention and Control Policy was approved along with the resolution 610 that established the national air quality standard. The standard was updated by the resolution 2254 of 2017, in which the maximum permissible PM_2.5_ levels of 50 and 25 µg·m^− 3^ were established for daily (24 h) and annual mean, respectively. Starting in 2018, the maximum permissible levels were more restrictive for an average exposure time of 24 h with limits of 37 µg·m^− 3^. These levels established in 2017 were still above the WHO 2005 AQG for PM_2.5_ daily (24 h) and annual mean of 25 and 15 µg·m^− 3^ respectively. The annual 2005 AQG was proposed to be achieved in 2030. The updated 2021 WHO AQG level for PM_2.5_ is 15 and 5 µg·m^− 3^ for daily and annual mean, respectively, and therefore the current national standard for PM_2.5_ annual mean corresponds to the new WHO interim target 2 [15].

Many of the world’s most developed countries measure PM_2.5_ concentrations through networks of monitoring stations, concentrated principally in urban areas. Although these data sources are valuable, in developing countries, air quality monitoring stations are scarce in urban areas, as well as far from mid-sized cities, suburban, and rural areas. Therefore, to obtain surface data of PM_2.5_ concentrations from these locations around the world, the results of air quality stations must be combined with satellite observations and information from global models [20].

Colombia has a national air quality network composed of 24 surveillance systems and 175 monitoring stations of which 92 monitored PM_2.5_ in 2019 [19] (IDEAM, 2021). Consequently, spatial PM_2.5_ resolution in all Colombian cities is limited, due to the lack of enough air quality stations. In addition, previous studies showed that the region contributes to biomass burning aerosol associated to PM [21], which occurs mainly in locations far from existing monitoring stations. Thus, the importance of using global models to estimate the exposure to PM_2.5_.

Recently, some products with high temporal and spatial resolutions from geostationary-orbit satellites have been available. Several studies assessed the relationship of PM_2.5_ and mortality, using satellite-derived estimations [22–28], mainly in the USA, Europe and Southeast Asia. Limited studies are available in South America [29–33].

For countries, estimating the avoidable mortality related to air pollution is central for decision making and for quantifying the potential health co-benefits that can be obtained with more strict air quality regulations. Consequently, the aim of the study was to compare estimates of spatiotemporal variations of surface PM_2.5_ concentrations in Colombia from 2014 to 2019 derived from two global air quality models, as well as to quantify the avoidable deaths at municipality level attributable to the long-term exposure to current and projected for 2030 Colombian standard for PM_2.5_ annual mean of 25 and 15 µg/m^3^, respectively.

## Materials and methods

### Population

Colombia is a country located in South America with an estimated total population of 49,395,678 inhabitants in 2019, with 29,221,754 over 25 years old, distributed in 1,122 municipalities and 33 departments, including the capital district [34]. According to this census, 51.2% of the total population are women and 71.8% of the population live in urban areas. Based on 2018 census, it is estimated that the total population in Colombia in 2030 will be 55,678,083 inhabitants.

### Data sources

#### PM_2.5_

According to the Colombian Institute of Hydrology, Meteorology, and Environmental Studies - IDEAM, ninety-two out of 1,122 municipalities regularly measure air quality in Colombia [19]. Large cities such as Bogota, Medellin, Bucaramanga, Cali, and Barranquilla have automatic air quality monitoring networks. On the other hand, medium-sized and smaller cities perform periodic manual measurements that are not readily available [35]. Because of the scarcity of surface measurements in the country, we retrieved PM_2.5_ concentrations at the surface level between 2014 and 2019 (as a measure of long-term exposure) from the global estimations of the Atmospheric Composition Analysis Group (ACAG) model and from the Copernicus Atmospheric Monitoring Service -CAMS- Reanalysis (CAMSRA). The ACAG is a global three-dimensional model that estimates surface concentrations by combining Aerosol Optical Depth (AOD) retrievals with GEOS-Chem chemical transport model and estimations are calibrated with global-ground based observations using a geographically weighted regression. We used the ACAG global surface PM_2.5_ estimations at 0.1° resolution which are freely available from the Washington University ACAG website as version V5.GL.01 [36]. CAMSRA uses four-dimensional variational data assimilation techniques that combines satellite observations with a global scale atmospheric model to produce aerosol and particle concentrations and mixing ratios of several gases at the surface and vertical gridded data [37, 38]. We obtained CAMSRA PM_2.5_ concentrations at the surface level over Colombia using the ECMWF Web API in Python provided at this platform [39]. We retrieved daily data at three hourly temporal resolutions and gridded at a 0.125° resolution (≈ 12 km) from January 1st, 2014, to December 31st, 2019. The PM_2.5_ concentrations were averaged per year and estimated at the centroid of each municipality by using the Inverse Distance Weighted (IDW) interpolation method from the nearest four retrieved CAMSRA concentrations [40]. Then, the results obtained from IDW interpolation were assessed statistically through the Spearman correlation coefficient (Rho) and Mean Bias (MB).

In order to evaluate the responsiveness of ACAG and CAMSRA PM_2.5_ data, we compared retrieved annual average PM_2.5_ concentrations with available ground-based measurements provided by IDEAM. We only used surface PM_2.5_ monitoring stations with more than 75% of measurements. We found that from 2014 to 2018, very few municipalities had enough information to compare with the models. Only in 2019, 28 municipalities reported PM_2.5_ measurements in 69 stations. Information from all the stations available in each municipality was averaged, and this average compared with ACAG and CAMSRA downloaded concentrations in each municipality using Pearson´s correlation coefficient and plotting the data with a linear regression line. We used Bland & Altman limits of agreement for estimating the mean differences between models estimations and ground concentrations and their 95% confidence intervals [41].

#### Mortality data, avoidable mortality and years of potential life lost estimation

The annual mortality data for 2014–2019 by municipality of residence and the codes from the International Classification of Diseases 10th version (ICD-10) were obtained from the public information system for social protection in Colombia (SISPRO, for its initials in Spanish) which compiles vital statistics validated from the National Department of Statistics (DANE, for its initials in Spanish) [42]. Population estimations by municipality and life expectancy for adults 25 years and older were obtained from the DANE [34].

We calculated the number of annual avoidable deaths related to the reduction of levels of PM_2.5_ concentration between 2014 and 2019 to accomplish the national standard of 25 µg/m^3^ implemented in Colombia since January 2011 and the 2030 projected national standard of 15 µg/m^3^ which corresponds to current WHO interim target 3. For this purpose, we calculated annual avoidable premature deaths for adults 25 years and older using the pooled effect estimates for total, natural and specific causes of mortality derived from meta-analyses of all international studies from Chen & Hoek [9] using Risk Ratios (RR) and selected international studies from Pope [43] using Hazard Ratios (HR), as shown in Table 1.

**Table 1:** Pooled effect estimates for PM_2.5_ exposure and mortality used for calculation of avoidable mortality

Mortality cause	ICD-10 codes	Pooled effect estimates^a^ (95% confidence interval)	Reference
Total	A00-Z99	1.08 (1.06–1.09)	Chen & Hoek, 2020 [9]
Total^b^	A00-Y98	1.08 (1.06–1.11)	Pope, 2020 [43]
Natural	A00-R99	1.08 (1.06–1.09)	Chen & Hoek, 2020 [9]
Circulatory system	I00-I99	1.11 (1.09–1.14)	Chen & Hoek, 2020 [9]
Respiratory system	J00-J99	1.11 (1.03–1.18)	Chen & Hoek, 2020 [9]
Cardiopulmonary	I00-I09, I11, I13, I20-I51, I60-I69, J09-J18, J40-J47	1.11 (1.08–1.14)	Pope, 2020 [43]
Respiratory malignant tumors	C30-C39	1.12 (1.07–1.16)	Chen & Hoek, 2020 [9]
Lung cancer	C33-C34	1.13 (1.07–1.20)	Pope, 2020 [43]
Ischemic Heart Disease	I20-I25	1.16 (1.10–1.21)	Chen & Hoek, 2020 [9]
Stroke	I60-I69	1.11 (1.01–1.34)	Chen & Hoek, 2020 [9]
Chronic Obstructive Pulmonary Disease	J40-J44, J47	1.11 (1.01–1.34)	Chen & Hoek, 2020 [9]
Acute Lower Respiratory Infections	J12-J18, J20-J22	1.16 (1.01–1.34)	Chen & Hoek, 2020 [9]

^a^Pooled effect estimates correspond to Risk Ratio (RR) for Chen & Hoek study and Hazard Ratio (HR) for Pope et al. study

^b^Total deaths excluding codes for Factors influencing health status and contact with health services (Z00-Z99) and codes for special purposes (U00-U99)

We used these RR/HRs as reference because there are no cohort studies to estimate the effect of long-term exposure on mortality available for Colombia and these studies are the most updated systematic review and meta-analyses of the effect of long-term exposure to PM_2.5_ on mortality based on results of 104 [9] and 75 [43] cohort studies around the world, respectively. The cohort studies included in these studies were conducted in North America, Europe, and Asia, with no studies from Africa, Central and South America; however, they included a wide range representation of PM_2.5_ concentrations that included mean annual levels in PM_2.5_ in Colombia. Using the RR/HRs derived from those meta-analyses, the number of annual avoidable deaths were calculated using the log-normal function expressed by Eq. (1) [44]:

1 $$\mathrm{Avoidable}\;\mathrm{deaths}\;\triangle Y\:=\:{\mathrm Y}_0\;\ast\;\mathrm P\mathrm{opulation}\;\ast\;\left(1-\mathrm e^{-\mathrm\beta\ast\triangle\mathrm{PM}}\right)$$

Where ΔY are the change in mortality expressed as avoidable deaths. Y_0_ is the baseline mortality rate for all causes or specific causes (2014–2019); Population is the exposed population, ΔPM is the annual PM_2.5_ concentration change from baseline (annual concentration obtained from models) to the Colombia standard of 25 µg/m^3^ which is the control scenario, and ß is the coefficient of theRR/HRs for an increase in PM_2.5_ concentration. This coefficient was calculated based on the RR/HRs calculated as pooled effect estimates from the studies from Chen & Hoek (2020) and Pope (2020) expressed according to Eq. (2) [45]:

2 $$\mathrm\beta\;=\;\ln\;(\mathrm{RR})\;/\;\triangle Q$$

where ΔQ refers to the PM_2.5_ concentration change that the studies used for RR/HRs estimation that is usually 10 µg/m^3^.

The baseline scenarios using the annual PM_2.5_ concentration at municipality level were obtained from satellite data from ACAG and CAMSRA models as described before. The control scenario was the Colombia standard of 25 µg/m^3^ implemented in Colombia since January 2011. Therefore, the annual number of avoidable deaths was zero for those municipalities with an annual PM_2.5_ concentration equals to or lower than 25 µg/m^3^. For those municipalities with annual PM_2.5_ concentration over 25 µg/m^3^, the avoidable deaths calculated represent the number of deaths that could be avoided if the national standard had been reached in that year. The total number of avoidable deaths 2014–2019 was calculated using the sum of the annual avoidable deaths for each municipality. The sum of avoidable deaths for municipalities of the same department were used to calculate annual and total avoidable deaths by department. The same procedure for calculating avoidable deaths was used with the projected 2030 national standard of 15 µg/m^3^ .

Furthermore, we calculated years of potential life lost (YPLL) due to nationwide total avoidable deaths by year for the period 2014–2019. First, we calculated age-group specific avoidable deaths as the product of age-group specific proportions of national deaths by the total number of avoidable deaths by year:

3 $$\varDelta {Y}_{}ij=\left(\frac{{D}_{ij}}{{D}_{i}}\right)\times \varDelta Yi$$

where i indexed the year of interest (2014, 2015, …, 2019), j indexed sixteen five-year age groups (25–29, 30–34, …, 95–99, ≥ 100 years), and D corresponded to the nationwide total number of deaths among individuals 25 years and older. Then annual YPLL was calculated as the sum of the products of age-group avoidable deaths and their mean life expectancies (LE):

4 $${YPLL}_{i}={\sum }_{=1}^{}\ 16\left(\varDelta {Y}_{ij}\times {LE}_{ij}\right)$$

## Results

### PM_2.5_ exposure estimations and comparison against monitoring data

**Fig. 1:**
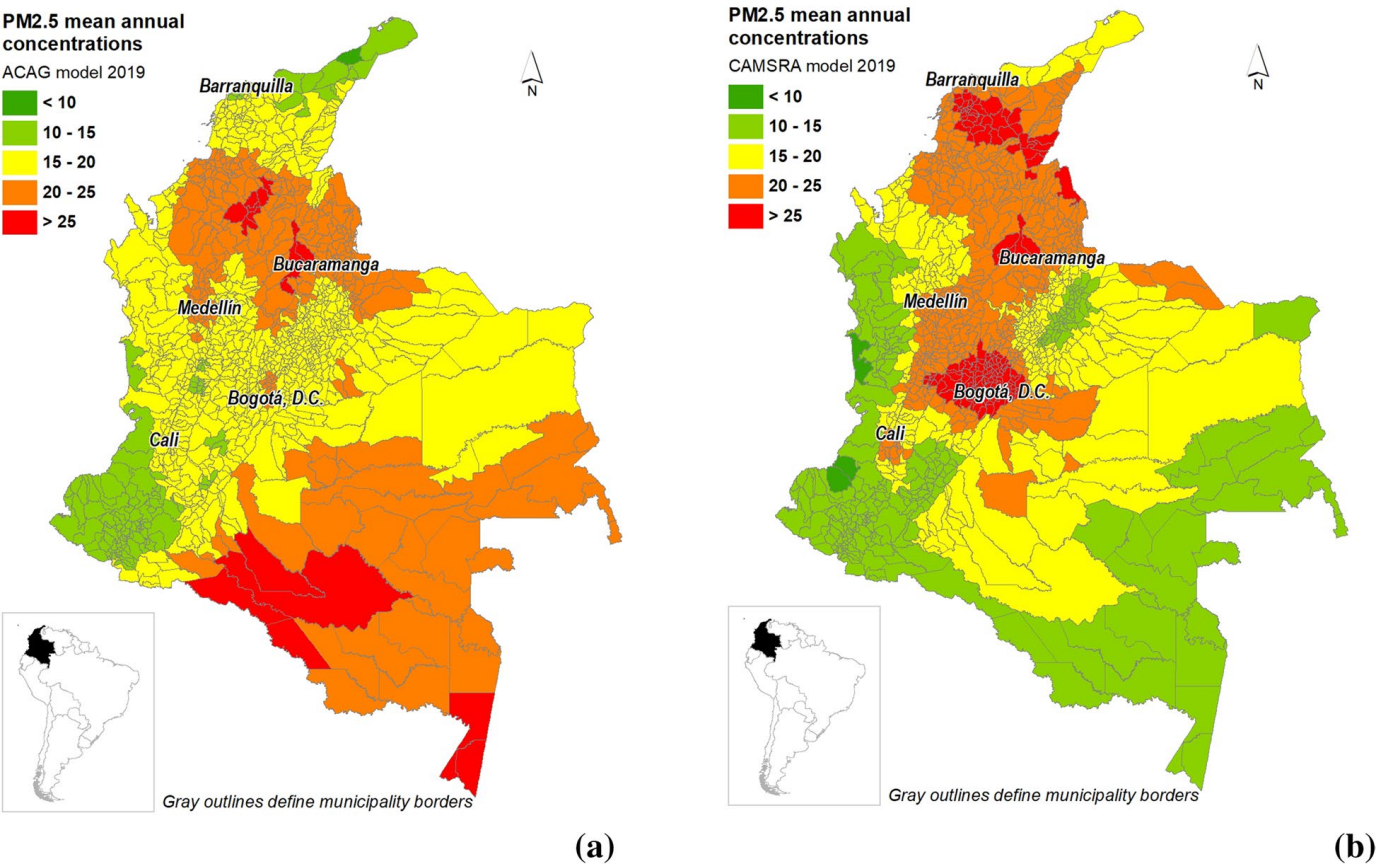
PM_2.5_ mean annual concentrations for 2019 at municipality level (**a**) Estimations based on ACAG model (**b**) Estimations based on CAMSRA model

**Fig. 2:**
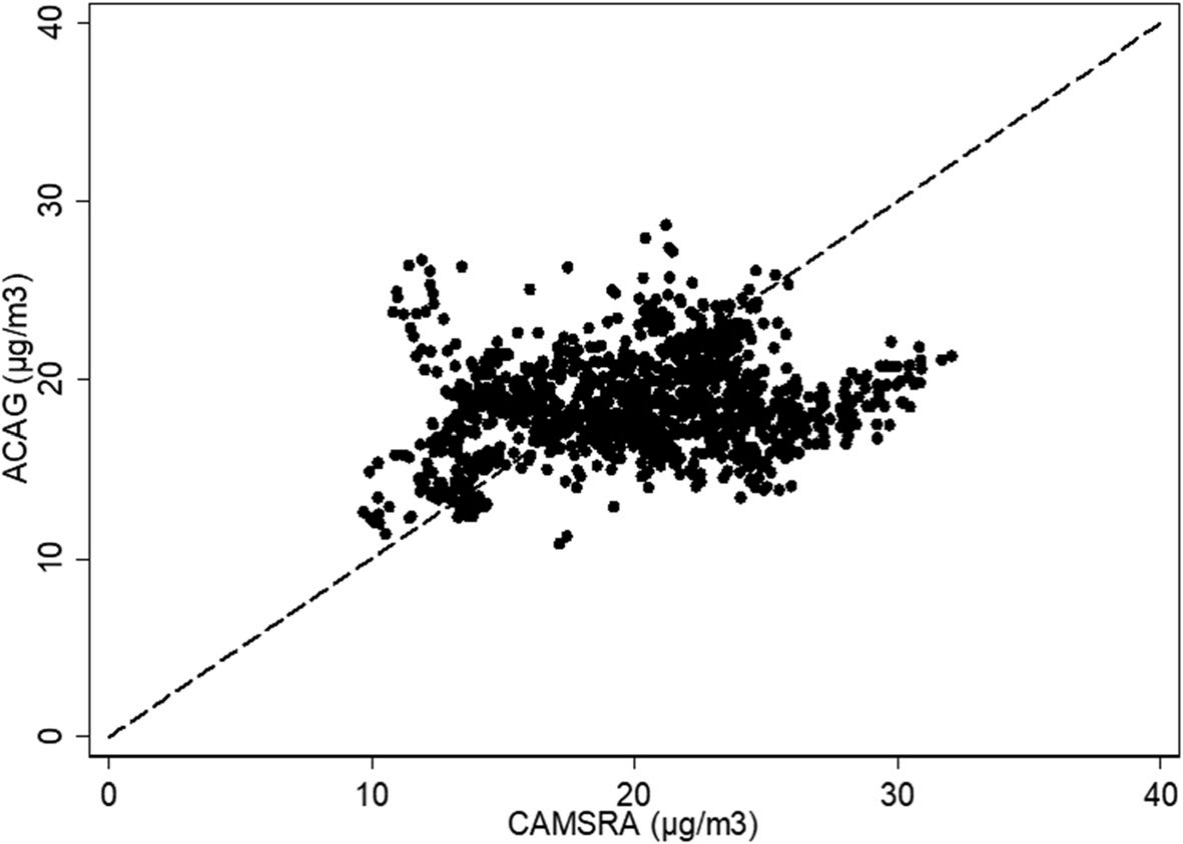
Comparison of surface PM_2.5_ mean annual concentrations for 2019 at municipality level based on estimations from ACAG and CAMSRA models

Figure 1 shows PM_2.5_ annual concentrations for 2019 at the municipality level retrieved from ACAG and CAMSRA models. There are significant differences in terms of the location and magnitude of the municipalities with the highest concentrations with both models. The ACAG model shows that the municipalities with the highest concentrations (above 20 µg/m^3^) are in the northern central and south part of the country and are mainly in Amazon area and its surrounding areas. On the contrary, CAMSRA shows that the highest concentrations are in the country’s center, mainly Bogotá and the municipalities at the west of this city, overlapping with the most densely populated region of Colombia (World Population Review, 2021. CAMSRA also showed high PM_2.5_ concentrations (> 25 µg/m^3^) in the north of the country and along part of the Caribbean coast, overlapping with highly populated municipalities. However, in that region, ACAG model reproduces lower concentrations that are between 15 and 20 µg/m^3^. The ACAG models reported PM_2.5_ concentrations over 20 µg/m^3^ over the eastern lowland, in the Amazon and the Orinoco basins. There are also some differences in concentrations over the time between 2014 and 2019, but in general ACAG models are consistent in estimating higher concentration in the north-central part and south part of the country and CAMSRA model are consistent in estimating higher concentrations in the central part of the country (See Supplementary figures S1-S2). Figure 2 shows the comparison of the estimated surface PM_2.5_ concentrations derived from ACAG and CAMSRA models for 2019 showing that CAMSRA exceeded estimations from ACAG model in 673 municipalities (60%) with a mean difference of 1,4 µg/m^3^.

**Fig. 3:**
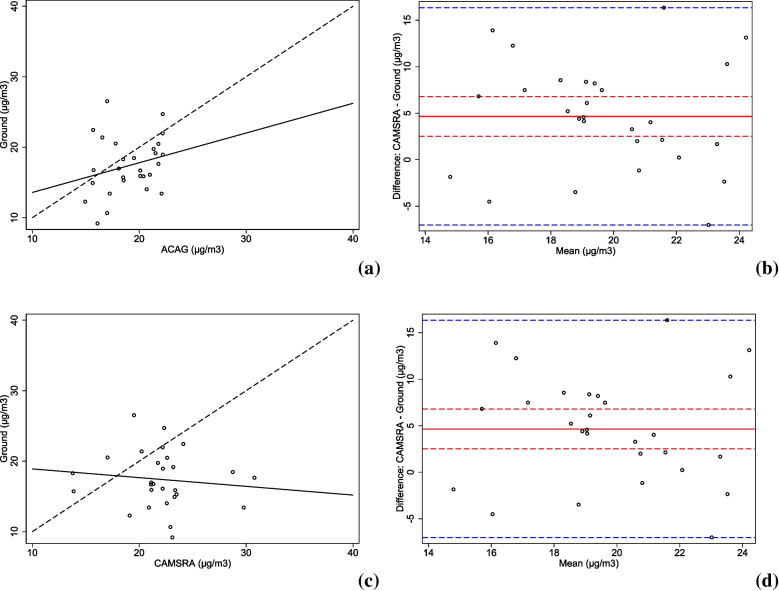
Comparison of ground- and model-based PM_2.5_ mean annual concentrations for 2019 in 28 cities. Scatter plot (**a**) and mean difference plot (**b**) compare ground-based concentrations with estimations based on ACAG model. Scatter plot (**c**) and mean difference plot (**d**) compare ground-based concentrations with estimations based on CAMSRA model. Note: In figures (**a**) and (**c**) the dotted lines represent the perfect correlation among measurements and the solid black lines represent the fitted regression line for the data. In figures (**b**) and (**c**) the dotted blue lines represent the ranges of the difference between model and ground data and the red lines represent the mean of the differences (solid red lines) and their 95% confidence intervals (dotted red lines)

We also evaluated the ability of ACAG and CAMSRA to reproduce surface PM_2.5_ concentrations. This evaluation compared surface data from 28 Colombian cities to the data obtained from the models. Figure 3 shows results from this comparison using yearly means in 2019 for ACAG and CAMSRA. Figure 3a) and c) show that ACAG and CAMSRA do not correlate well with ground-based measurements (ρ = 0.25, p = 0.193 and ρ=-0.12, p = 0.558, respectively) and overestimate annual ground-based average PM_2.5_ concentrations from most cities (Fig. 3b and d): for ACAG model in 22/28 (78.6%), mean bias = 1.7 µg/m^3^; 95%CI: 0.1–3.3 and for CAMSRA model in 22/28 (78.6%), mean bias = 4.7 µg/m3; 95%CI: 2.4–6.9. Both ACAG and CAMSRA overestimate ground-based concentrations of PM_2.5_ for Bogota, the largest city in Colombia (about 6% and 56%, respectively), and Medellin, the second largest city (about 8% and 10%, respectively), whereas for Cali, the third-largest city, both models underestimated ground-based measurements (23% and 5%, respectively).

Using the ACAG model, there were a total of 10 (1%) municipalities exceeding the level of the national standard of 25 µg/m^3^ in 2014, 27 (2,4%) in 2015, 40 (3,6%) in 2016, 15 (1,3%) in 2017, 10 (1%) in 2018, and 18 (1,6%) in 2019. On the other hand, using the CAMSRA model, there were a total of 169 (15,1%) municipalities exceeding the level of the national standard in 2014, 238 (21,2%) in 2015, 233 (20,7%) in 2016, 81 (7,2%) in 2017, 81 (7,2%) in 2018, and 150 (13,4%) in 2019. For the same year, the PM_2.5_ ground-based concentrations for de 28 cities showed that only one municipality exceed the annual standard. Therefore, the ACAG model showed a better estimation of ground-based concentrations compared to CAMSRA model. For the scenario with annual PM_2.5_ concentration standard of 15 µg/m^3^, using the ACAG model, there were 97 (8.6%) municipalities in 2014 that comply this limit, 94 (8.4%) in 2015, 93 (8.3%) in 2016, 116 (10.3) in 2017, 172 (15.3) in 2018, and 127 (11.3%) in 2019.

### Estimation of avoidable mortality

**Fig. 4:**
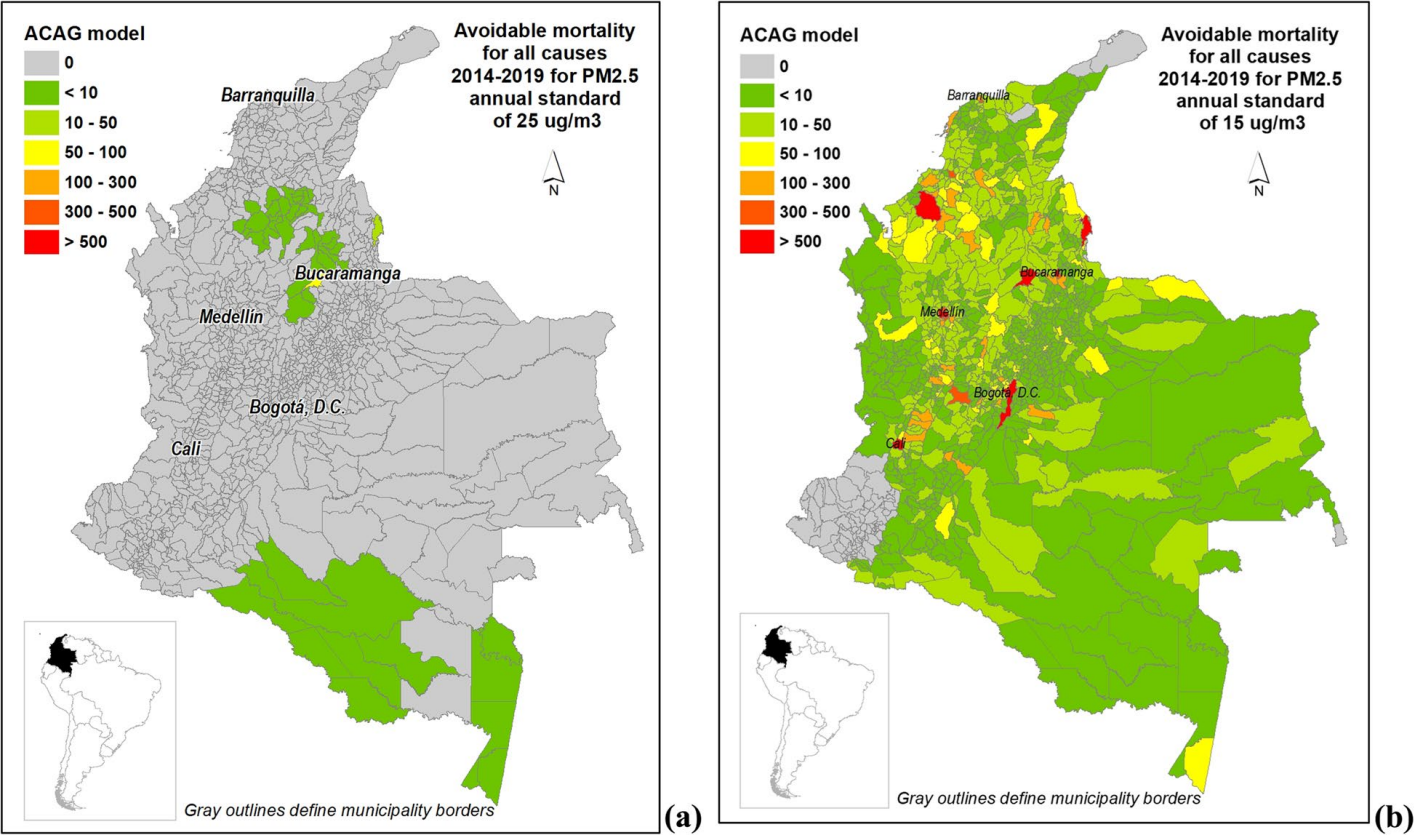
Avoidable mortality for all causes derived from estimations of annual surface PM_2.5_ concentrations based on ACAG model by municipality, Colombia, 2014–2019 (**a**) for national standard of 25 µg/m^3^ (**b**) for international interim target of 15 µg/m^3^

Based on the results from the comparison of both air quality models with surface data, we chose the ACAG model the estimation of the avoidable mortality. Using PM_2.5_ estimates at municipality level from ACAG model, the total number of avoidable deaths during 2014–2019 was 142 for current PM_2.5_ annual standard of 25 and 34,341 for the projected 2030 annual standard of 15 µg/m^3^. Table 2 shows the number of avoidable deaths by year for the current and projected PM_2.5_ annual standard of 25 and 15 µg/m^3^ using the ACAG model. Figure 4 shows the avoidable mortality for all causes using ACAG model by municipality for 25 and 15 µg/m^3^ annual mean concentrations.

**Table 2:** Number of avoidable deaths using national standard and international interim target 3 as control scenarios, by mortality cause using the ACAG model for surface PM_2.5_ concentration estimations, Colombia 2014–2019

Mortality cause	National standard of 25 µg/m^3^								WHO interim target 3 of 15 µg/m^3^							
	2014	2015	2016	2017	2018	2019	Total	Rate per million	2014	2015	2016	2017	2018	2019	Total	Rate per million
All causes	13.82	33.90	63.97	4.69	5.28	20.35	142.01	5.18	6373.93	6277.51	6250.06	5416.70	4157.36	5865.66	34341.22	1253.24
Natural	11.56	28.36	58.49	3.96	4.63	19.02	126.01	4.60	5446.11	5332.35	5811.13	5058.35	3858.30	5460.55	30966.80	1130.09
Circulatory	6.10	15.13	24.88	2.05	2.99	9.70	60.85	2.22	2323.20	2302.94	2212.26	1890.48	1446.12	2016.85	12191.85	444.92
Respiratory	2.90	6.88	14.95	0.92	0.79	4.47	30.92	1.13	1473.80	1414.06	1415.53	1304.40	966.25	1412.04	7986.09	291.44
Cardiopulmonar	6.54	16.02	26.83	2.29	3.21	10.02	64.90	2.37	2429.83	2505.81	2411.67	2067.68	1576.37	2203.17	13194.53	481.52
Malignant respiratory tumors	0.09	0.23	0.20	0.00	0.00	0.07	0.59	0.02	41.95	45.10	39.56	36.39	29.67	40.27	232.93	8.50
Lung cancer	0.08	0.25	0.19	0.00	0.00	0.06	0.58	0.02	39.08	42.52	36.88	34.42	29.27	38.39	220.57	8.05
Ischemic heart disease	4.28	8.91	13.23	0.61	1.38	6.41	34.81	1.27	1188.18	1177.65	1128.39	996.18	776.75	1168.67	6435.82	234.87
Stroke	0.32	1.16	1.83	0.20	0.27	0.93	4.71	0.17	214.78	209.32	196.22	179.15	142.83	191.66	1133.96	41.38
COPD	0.21	0.18	0.35	0.07	0.06	0.16	1.03	0.04	57.52	46.99	42.73	36.20	26.59	38.02	248.05	9.05
ALRI	0.44	1.62	3.67	0.23	0.26	1.08	7.30	0.27	153.63	306.26	303.61	266.17	203.00	292.29	1524.96	55.65

*ACAC* Atmospheric Composition Analysis Group

*ALRI* Acute Lower Respiratory Infections

*COPD* Chronic Obstructive Pulmonary Disease

*WHO* World Health Organization

For comparison, the total avoidable deaths estimated for the 28 cities with data from monitor stations in 2019 showed only 6 avoidable deaths from the municipality of Yumbo near to the city of Cali. The total avoidable deaths estimated with CAMSRA model were on average 52 times higher than the estimation using the ACAG model (7,368 deaths; 268,9 deaths per million people over 25 years old) (See supplementary table S1). Large differences are explained mainly because annual PM_2.5_ estimations using CAMSRA were higher and over the national standard for more municipalities, and particularly for Bogotá and surrounded municipalities. Considering the current standard of 25 µg/m^3^ as reference and the ACAG model, the municipalities with the highest total avoidable deaths for all causes were Barrancabermeja (57), San José de Cúcuta (19), both locate at the northeast of the country, and Leticia (7), the capital of the department of Amazonas. Avoidable mortality by department using ACAG model for PM_2.5_ exposure estimation for annual standard of 25 and 15 µg/m^3^ and estimations by municipality for annual standard of 15 µg/m^3^ are presented in Supplementary material (Tables S2-S3).

**Fig. 5:**
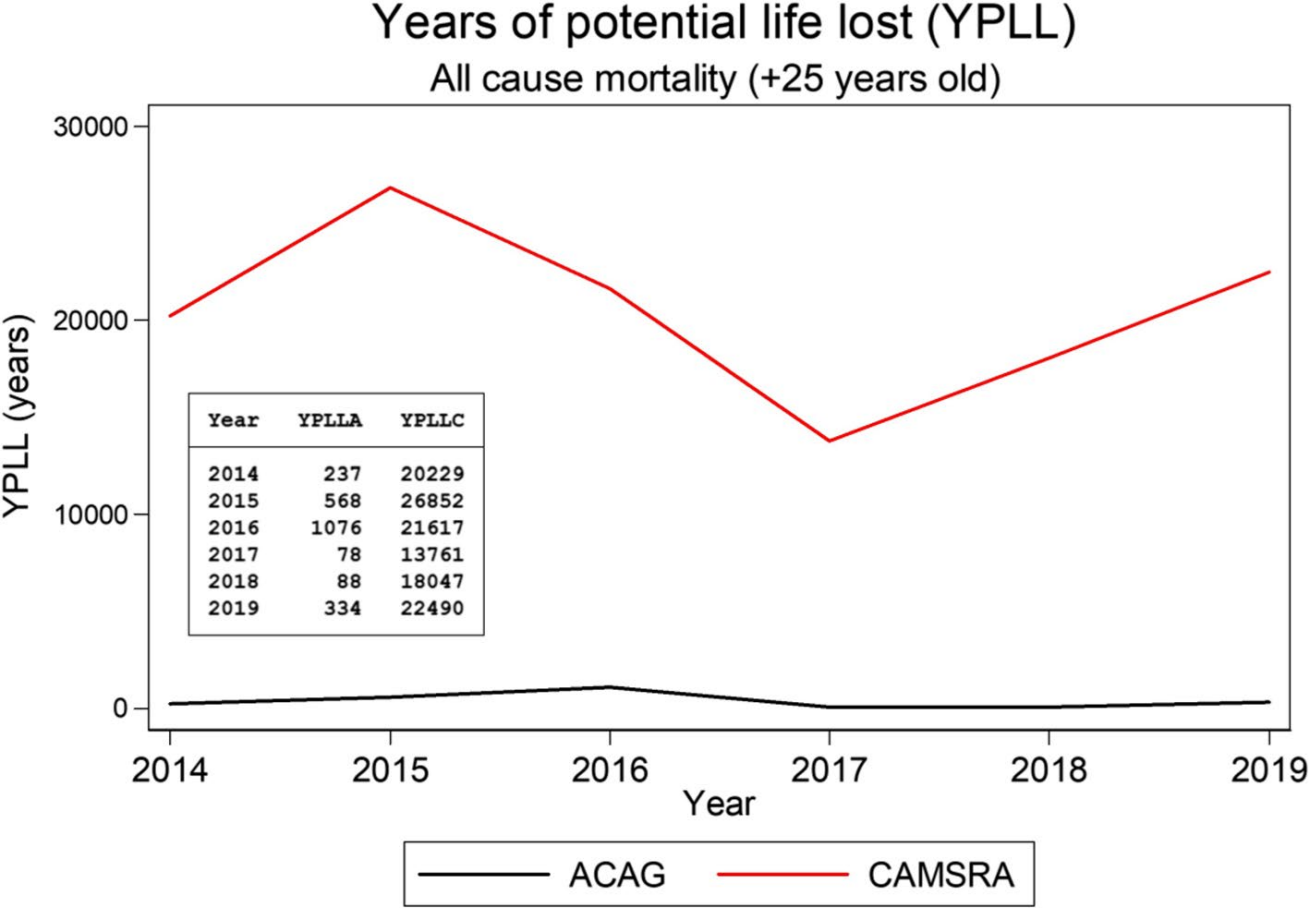
Years of potential life lost attributable to exposure to annual PM_2.5_ concentrations above 25 µg/m^3^ as estimated using ACAG (YPLLA) and CAMSRA (YPLLC) models

Avoidable deaths related to cardiopulmonary causes accounted for 45% and 38% of the total preventable deaths using the ACAG model for annual mean concentrations of 25 and 15 µg/m^3^, respectively. For the estimations using the 15 µg/m^3^ level as control reference, the ischemic heart disease represented 18% of total preventable deaths, while acute lower respiratory infections represent 4%, and lung cancer represented less than 1% of total avoidable deaths. Finally, the calculated total YPLL due to all-cause mortality for the current PM2.5 annual standard of 25 µg/m^3^ for the period 2014–2019 were 2,381 and 122,996 years based on ACAG and CAMSRA PM_2.5_ estimations, respectively (Fig. 5). In accordance with the number of avoidable deaths, YPLL from ACAG estimations had lower mean and annual variation than YPLL from CAMSRA: 397 years (range: 78 − 1,076) and 20,499 (range: 13,759 − 26,851), respectively.

## Discussion

Our study estimated the avoidable mortality due to long-term exposure to PM_2.5_ in Colombia during 2014–2019 having as control scenarios the current national annual standard of 25 µg/m^3^ and the projected standard for 2030 of 15 µg/m^3^ which correspond to the current WHO interim target 3. The estimated avoidable deaths were calculated at municipality level as the number of deaths that could be avoided if the national standard for PM_2.5_ annual mean of 25 µg/m^3^ have been met and the avoidable deaths if the PM_2.5_ annual mean of 15 µg/m^3^ have been implemented. Estimations of attributable deaths to PM_2.5_ exposure differed depending on the global air quality model used for estimating the ground levels at municipality scale: More accurate surface PM_2.5_ concentration estimations for Colombia, and therefore more accurate estimated avoidable mortality were obtained using ACAG model.

Ground-based monitoring is ideal due to its high accuracy. Nevertheless, it is not feasible with geographic coverage. The few PM_2.5_ air quality networks in developing countries may limit our ability to accurately assess human exposure to PM_2.5_ since measured concentrations may vary with increasing distance from the monitoring station. For this reason, advances are emerging in using ground-based data jointly with land use regression (LUR) and air quality models, satellite information, and low-cost sensors for improvement of air quality estimated data. This combination of techniques may provide better spatial coverage, although information on temporal coverage is still being studied [46]. These developments are especially important if used to estimate personal exposure and variability within a city.

There were differences in estimations of ground concentrations using ACAG and CAMRA models, particularly in the Amazon and the Orinoco basins. Although this country’s area has less than 3% of the population, this region is affected by seasonal wildfires between January and April that produce large amounts of PM_2.5_ [21]. Differences between estimated data from models and ground monitoring information may be due to uncertainty of emission inventories or the incorrect representation of the meteorology in the region. Validation of air quality models with regional inventories is common for the United States, Canada, México, Europe, and East Asia [47, 48], although not for Colombia or other developing countries. For CAMSRA, modeling inconsistencies due to cloud interference in the Amazon and South America, or some smoke episodes may overestimate values in some cases [49]. For ACAG, the MODIS data used in the Global Fire Emission Database (GFED) inventories is too coarse to detect the small and transient burning fires or other local emissions; thus, estimated values from both models may vary from ground-based monitoring air quality stations [50]. During the last years, events of Saharan dust intrusion to the Andes are rather scarce but have affected air quality in Medellín, Bogotá, and North of Colombia; increased by regional biomass burning in the Orinoco basin and the Magdalena Valley [51, 52]. Despite the substantial improvement of spatial resolution of atmospheric emission inventories in most developed countries, few emission datasets have been addressed for temporal profiles [53].

As explained above, model performance depends on the uncertainty of the emission inventories and meteorology. Differences between estimated data from models and ground monitoring information may be due to regional emission inventories, modeled meteorology uncertainty and accuracy of the satellite- retrieved total column aerosol optical depth (AOD). Van Donkelaar et al. [36] studied monthly global estimates of fine particulate matter and their uncertainty for ACAG model, were results pointed the obtained largest uncertainties over the relatively under-monitored regions of Africa, Latin America and Asia. For example, uncertainties in Andean and Tropical region of Latin America ranges from 3.4 to 4.4 µg/m^3^ and 0.5–2.3, respectively. For CAMSRA model, mostly all profile are based on western European data and therefore the source of uncertainty for other world regions [53]. Using the same spatial resolution for both models (around 10 Km x 10Km), the ACAG model seem to capture better emission from Amazonian region and more precise estimation in other regions compared to CAMSRA model.

Using the ACAG model, for 2019 the exceedances were more frequently present over the current national standard in municipalities located in the departments of Antioquia, Córdoba, Santander, and particularly in departments at south of the country with lower population density, and therefore they account for few total avoidable deaths. In contrast, municipalities in the department of Atlántico, located in the Caribbean region, showed no exceedances using the ACAG model for any of the year with no contribution to the total avoidable deaths under the current annual standard. Estimated avoidable deaths in Colombia for PM_2.5_ annual average scenario of 15 µg/m^3^ using ACAG model were concentrated in particularly in Bogotá, Medellín, Cali, and Bucaramanga, regions with high population density.

In Colombia, the national study of economic valuation of environmental degradation estimated that 8.030 deaths in the population over 44 years in 2015 were attributable to urban air pollution; 92% (7.362 deaths) were related to cardiopulmonary diseases and 8% (668 deaths) were related to lung cancer. Overall, the economic valuation of attributable deaths was estimated at 10,6 billion pesos. This study estimated PM_2.5_ concentration based on PM10 reports of the national monitoring system and included only the population from 21 regions where air quality surveillance systems were in place, which included nearly half of the country’s population (DNP, 2018). The national study of burden of environmental diseases estimated 15,681 deaths attributable to air pollution in 2016 following the methodology of the GBD study 2015 and used PM_2.5_ data from available monitoring stations; the deaths were mainly related to IHD (rate 290.15 though 100,000 population) and COPD (143.99 per 100,000 population). The attributable fraction of IHD and COPD due to PM_2.5_ were estimated at 15.8% and 17.5%, respectively, compared to 2.8% and 4% due to indoor pollution (INS, 2018). Differences in attributable deaths in these national studies are probably explained by the population covered, sources and estimation of PM_2.5_ levels, the causes of deaths included, and exposure-response curves used.

Our study adds to previous studies on avoidable mortality related to ambient air pollution in Colombia as we included all municipalities of the country by using PM_2.5_ estimates from two different air quality global models, and used the up-to-date global estimations of pooled-effect measures (RR/HRs) derived from hundreds of cohort studies conducted around the world, including developing countries with a wide range of PM_2.5_ annual mean levels [9, 43]. However, it is important to note that estimates of our study correspond to the deaths attributable to the excess of PM_2.5_ exposure over the current and projected 2030 national annual standard and they did not represent the total deaths attributable to PM_2.5_ exposure during the study period; therefore, our estimates cannot be compared directly with estimates of total deaths from the national studies mentioned above. It is also important to note that our analysis is concerned only with long-term exposure to PM_2.5_ represented by an average exposure over six years (2014–2019) and therefore our results are not comparable with those from analysis of short-term effects that usually use days as time variable for the analysis.

National standards differ by country and Colombian annual standard for PM_2.5_ is the highest in the region of the Americas. The WHO global AQG were updated in September 2021 being one of their objectives to “provide interim targets to guide reduction efforts towards the ultimate and timely achievement of the AQG levels for those countries that substantially exceed the AQG levels” (WHO, 2021). According to the updated WHO air quality guideline, the current Colombian PM_2.5_ annual standard of 25 µg/m^3^ corresponds to the new interim target 2. In Brazil and Chile, the current national PM_2.5_ annual mean standard is 20 µg/m^3^ and for Ecuador the standard is 15 µg/m^3^ which corresponds to the new interim target 3 [54]. According to the new WHO guidelines all countries need to strengthen their effort in reducing PM_2.5_ sources and emissions and review their national standards in a route to decrease the morbidity and mortality attributable to ambient air pollution.

There are limited studies assessing the amount of avoidable mortality at a nation-wide scale in South America and most of them have been conducted in Brazil. Andreão et al. [29] studied 102 cities in the southeast region of Brazil by the estimation of daily PM levels using satellite data and mortality that would be avoided if they comply with WHO air quality guidelines. Results of this research showed that particle concentrations exceeded WHO guidelines by 8 to 12 times. Recently, Andreão et al. [44] assessed avoidable mortality during 2014–2018 including 5570 Brazilian cities using ACAG model for estimating PM_2.5_ annual mean; the total avoidable deaths were estimated in 1,335 (10,7 per million population over 25 years old) for a target of 20 µg/m^3^ and 48,700 deaths (389,6 per million population over 25 years old) for a target of 10 µg/m^3^. Despite the wide coverage of Brazilian cities, the results of this study showed lower estimates of total avoidable deaths compared to our study in Colombiabased on the ACAG model when comparing with the intermediate standard of 10 µg/m^3^. It is important to note that for 2014 and 2018 in Brazil there were no cities exceeding this standard and in 2015 exceedances occurred only in 2.7% of the cities, which imply a high compliance to the standard. Similarly, exceedances above the standard of 25 µg/m^3^ using the ACAG model ranged between 1% and 4% of the cities during the same years in Colombia, which might explain the low number of estimated attributable deaths.

Results of Brazilian studies also showed that exposures to these fires induces acute health disorders [29, 44]. Colombia, similar to Brazil, suffers the effects of the high levels of particles emitted from wildfires with significant fire activity [27]. Also, burns over the Amazonia in South America and grassland plains in Northern South America during the dry season fires and the Orinoco River basin deteriorate air quality in highly populated urban centers hundreds of kilometers away from the sources [21].

Colombia updated its National Determined Contribution (NDCs) in 2020 having as its central goals the reduction in greenhouse emissions by 51%, and the reduction of black carbon emission by 40% by 2030 (compared to 2014 levels) [55]. The Colombian NDCs are aiming to achieve the national goals for the Sustainable Development Goals (SDGs) which were reinforced as political goals for all countries in the Glasgow Pact 2021 [56]. It is also now well recognized that countries need to make a joint effort in reducing air quality and climate change strategies as they are strongly related.

Countries need to assess the effects of air pollution on human health as a strategy to advocate for more restrictive air quality control and climate change mitigation goals. In this regard, quantification of mortality attributable to ambient air pollution is a key indicator of burden of disease due to air pollution. Seen in the benefit-point of view the total of avoidable deaths are the total deaths that might be prevented if PM_2.5_ standard had been accomplished and more restrictive standard is introduced. WHO recommends the quantification of the health co-benefits related to reduction in air pollutants concentrations as part of their national NDCs [57]. Quantifying avoidable deaths lets the government and citizens assign a value to the magnitude of the long-term air pollution effects, and define local, regional, and national goals and resources allocation. Moreover, distribution of avoidable deaths might differ by geographical area and therefore spatial distribution of avoidable deaths at municipality level, as presented in this study, informs local governments and communities for tailored local air quality planning.

Strengths of this study include the use of two global air quality satellite-based models to estimate PM_2.5_ ground concentrations at municipality level and the comparison of both model estimates with air quality monitoring data. This is the first study in Colombia and one of the limited studies in South America to assess the avoidable mortality related to PM_2.5_ long-term exposure using satellite-based global air quality models. Estimates differed widely between CAMSRA and ACAG models but coincide in showing elevated levels of PM_2.5_ not only in large urban areas but also in some medium-size and small municipalities. This geographical distribution of aerosols has been reported in other studies for Colombia, which showed the influence of biomass burning in small and large municipalities [58, 59]. The biomass burning along with a wide variability in meteorological conditions across the country might explain to some extent the difference between both model estimates and between model estimates and ground levels. The main difference between ACAG and CAMSRA is the input information used, especially the emissions datasets. ACAG uses the GFED4 biomass burning emission inventory as input information to quantify pollutants produced from this source, while CAMSRA uses the GFASv1.2 data set. A recent study compared these two and other data sets and found significant discrepancies between biomass burning emissions reported (Pan et al. 2020). Therefore, this is one important reason for the PM_2.5_ concentration differences found in this study. Further research should improve emission estimations from biomass burning and identify other sources of uncertainty. In addition, our study estimated the number of avoidable deaths using updated pooled-effect estimates from the most recent global meta-analysis of cohorts assessing long-term effects on mortality. Although this meta-analysis did not include cohorts from Colombia or other countries in South America, they do include studies from different regions of the world including countries in South Asia and China which cover a wide range of long-term PM_2.5_ exposure. Therefore, estimations of RR/HRs for mortality from these global studies (Chen and Hoek, 2020; Pope et al., 2020) are the best available estimate for assessing risks for mortality related to long-term PM_2.5_ exposure.

One important limitation of our study is that only 28 municipalities (2.5%) had available PM_2.5_ data from ground air quality monitoring systems, which limited the comparison with estimates from global satellite-based models. Despite the small percentage of municipalities represented, the municipalities with available data account for most of the large cities and the municipalities with coal extraction in the country [19]. However, the scarce data from the Orinoquia and Amazon region do not represent concentrations derived from these large mostly rural areas of the country where biomass burning occur. This limitation, however, do not affect the results for estimations of avoidable deaths derived from global models.

## Conclusion

Comparison of two global air quality satellite-based models for estimating surface PM_2.5_ concentrations during 2014–2019 at municipality scale in Colombia showed important differences. Compared to surface data from monitoring stations from 28 cities in 2019, ACAG model estimates of PM_2.5_ surface concentrations were more accurate compared to CAMSRA model.

We estimated a total of 142 and 34,341 avoidable deaths during 2014–2019 due to long-term exposure to PM_2.5_ exceeding the current and projected national standard annual mean of 25 and 15 µg/m^3^ using estimations based on the ACAG global air quality model. For both scenarios, the cardiopulmonary diseases, and particularly the IHD, accounted for most of the attributable deaths due to PM_2.5_ excess of exposure. These numbers represent the range of total number of deaths that could be avoided if the current national standard for PM_2.5_ annual mean of 25 µg/m^3^ have been met and more restrictive standard have been implemented. Estimates of avoidable deaths related to PM_2.5_ excess of exposure at municipality level might inform national and local decision makers about priority air quality interventions and support the health co-benefits of making more restrictive air quality regulations.

## Supplementary Information

**Additional file 1:** **Figure S1.** Estimations of surface PM_2.5_ mean annual concentrations at municipality level based on the ACAG model, Colombia, 2014-2019. **Figure S2.** Estimations of surface PM_2.5_ mean annual concentrations at municipality level based on CAMSRA model, Colombia, 2014-2019.

**Additional file 2: Table S1.**  Number of avoidable deaths using national standard of 25 ug/m^3^ as control scenario using exposure estimations from CASAG and CAMSRA global air quality models for surface PM_2.5_, Colombia 2014-2019. **Table S2.** Number of avoidable deaths by departments using national standard and international interim target 3 as control scenarios by mortality cause using ACAG model for surface PM2.5 concentration estimations, Colombia 2014-2019. **Table S3.** Number of avoidable deaths by municipality using WHO interim target 3  of 15 µg/m^3^ as control scenario using exposure estimations from ACAG global air quality model, Colombia 2014-2019.

## Data Availability

The datasets used and/or analysed during the current study are available from the corresponding author on reasonable request.
