# Supplementary material for: Avoidable mortality due to long-term exposure to PM2.5 in Colombia 2014–2019
Source: Environ Health. 2022 Dec 24;21:137. doi: 10.1186/s12940-022-00947-8 (PMC9789551; doi:10.1186/s12940-022-00947-8)
Supplement: Supplementary file 1 — Additional file 1: Figure S1. Estimations of surface PM2.5 mean annual concentrations at municipality level based on the ACAG model, Colombia, 2014-2019. Figure S2. Estimations of surface PM2.5 mean annual concentrations at municipality level based on CAMSRA model, Colombia, 2014-2019. [file 12940_2022_947_MOESM1_ESM.docx]

**Figure S1.** Estimations of surface PM_2.5_ mean annual concentrations at municipality level based on the ACAG model, Colombia, 2014-2019

| 1. 2014   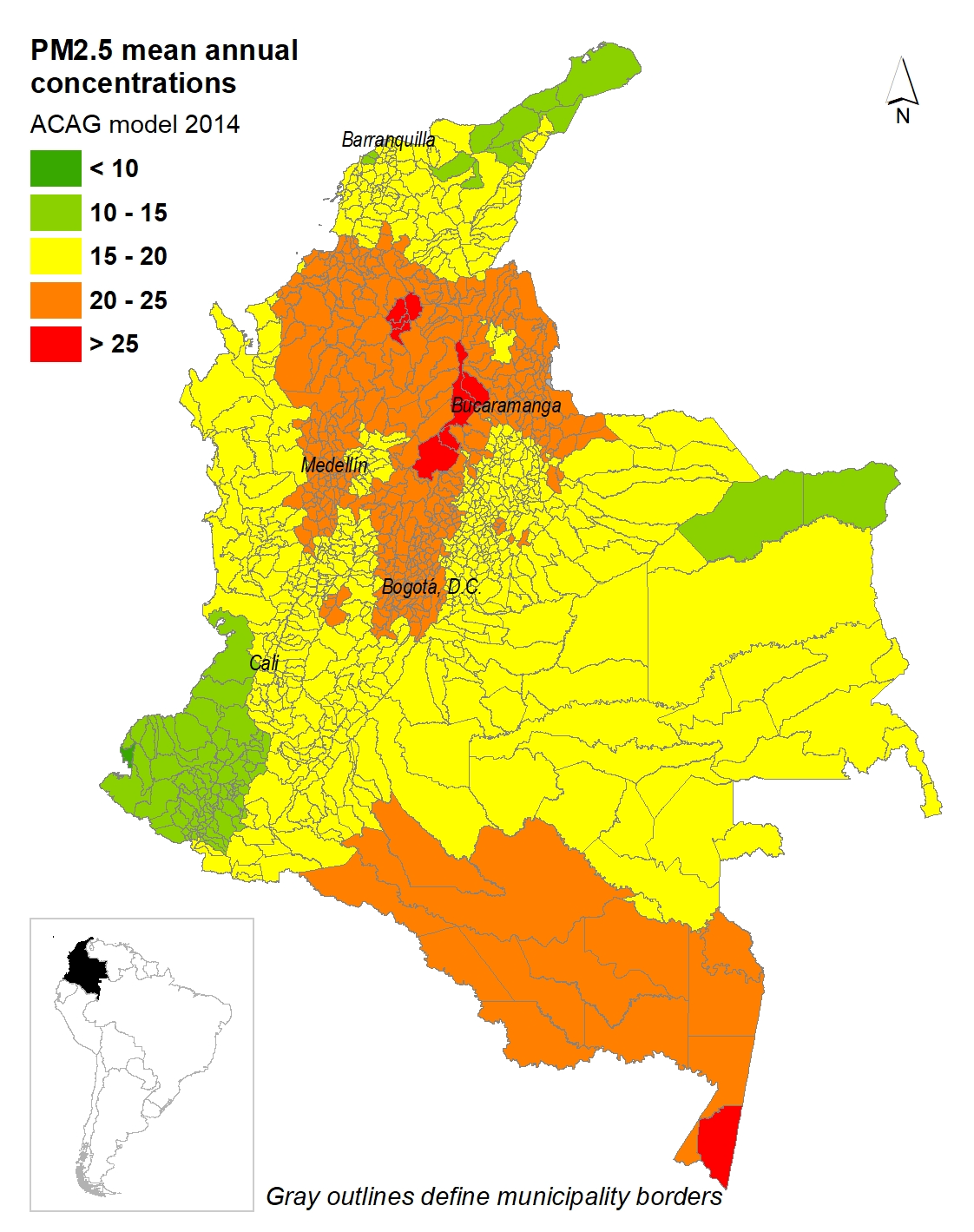 | 1. 2015   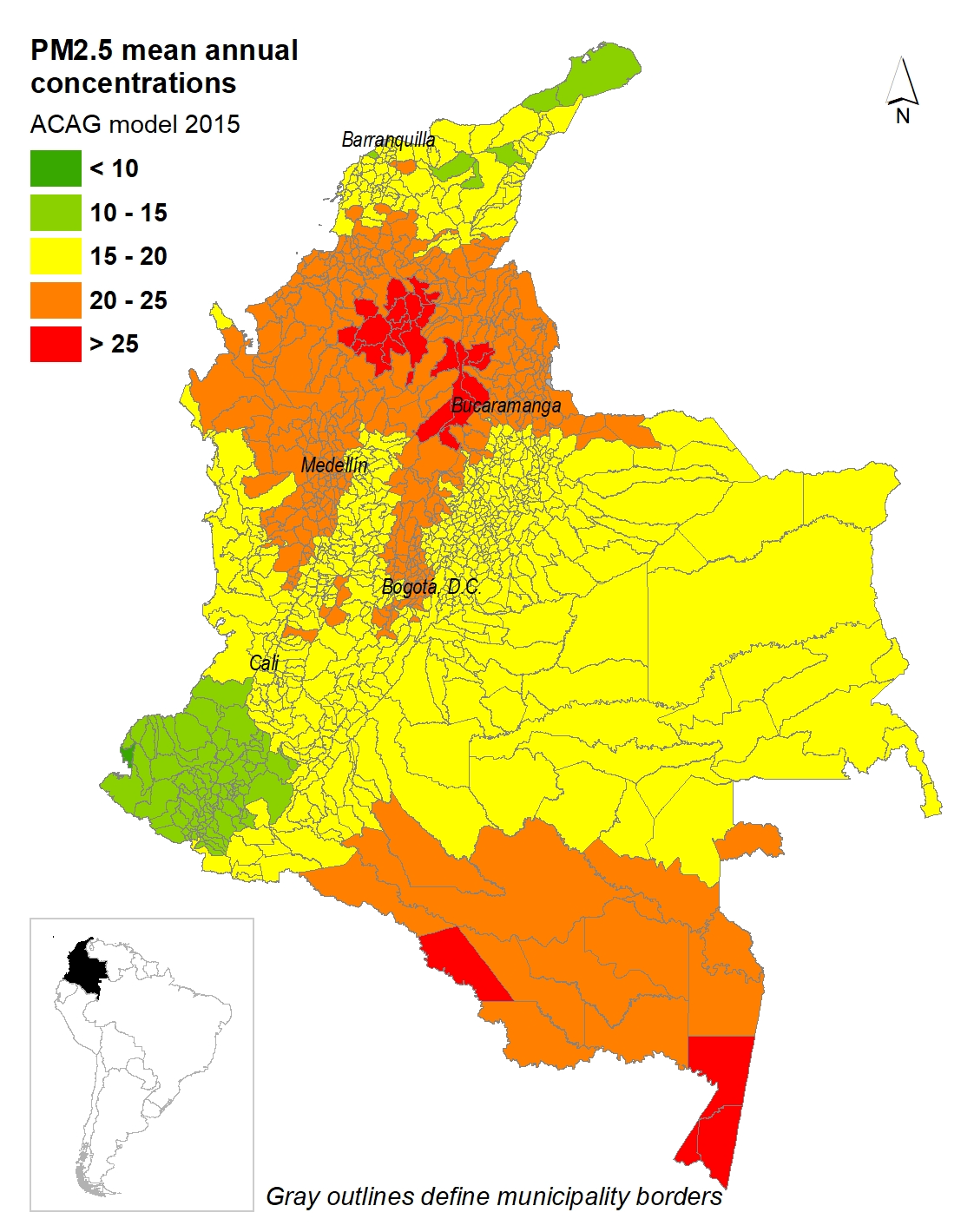 |
| --- | --- |
| 1. 2016   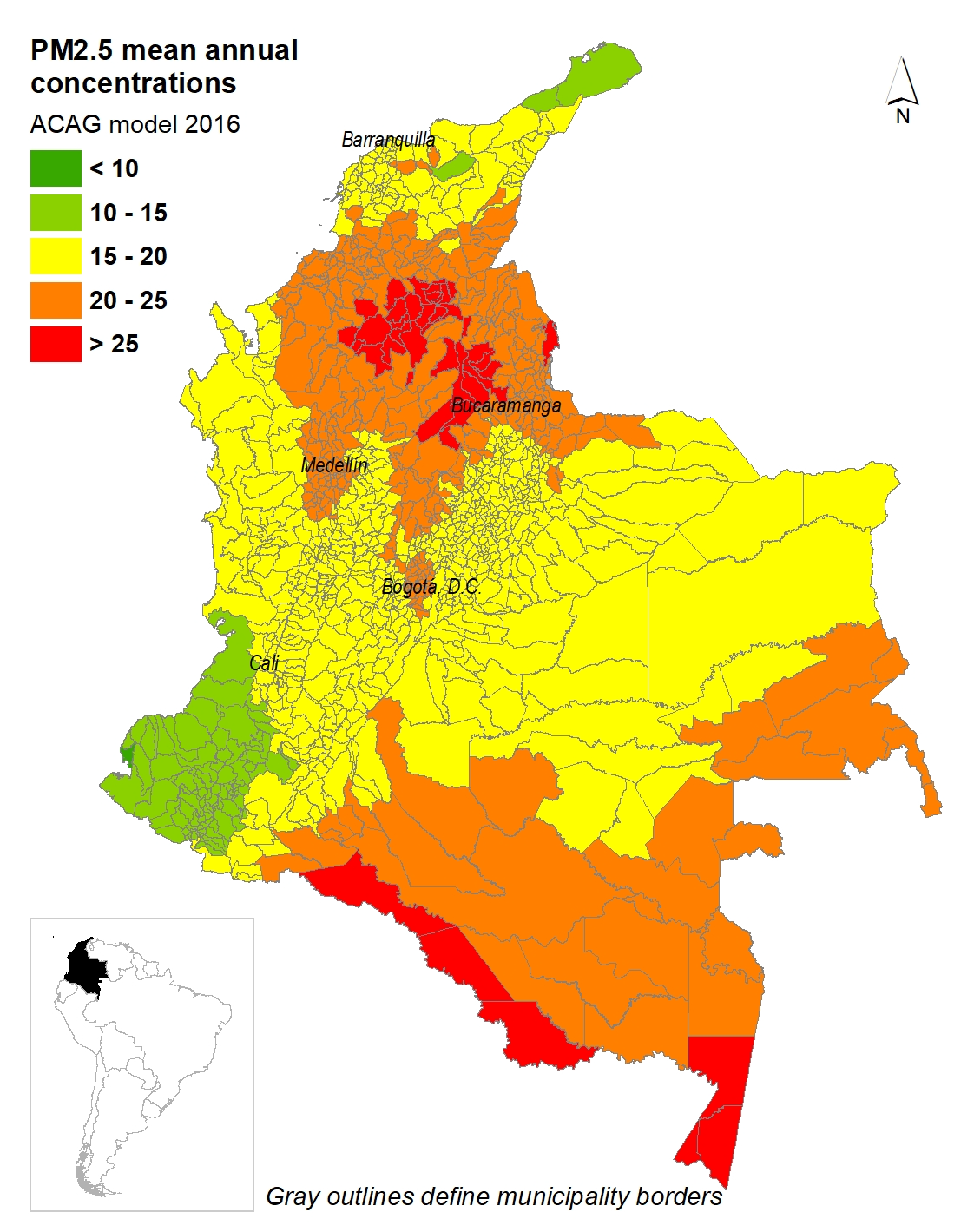 | 1. 2017   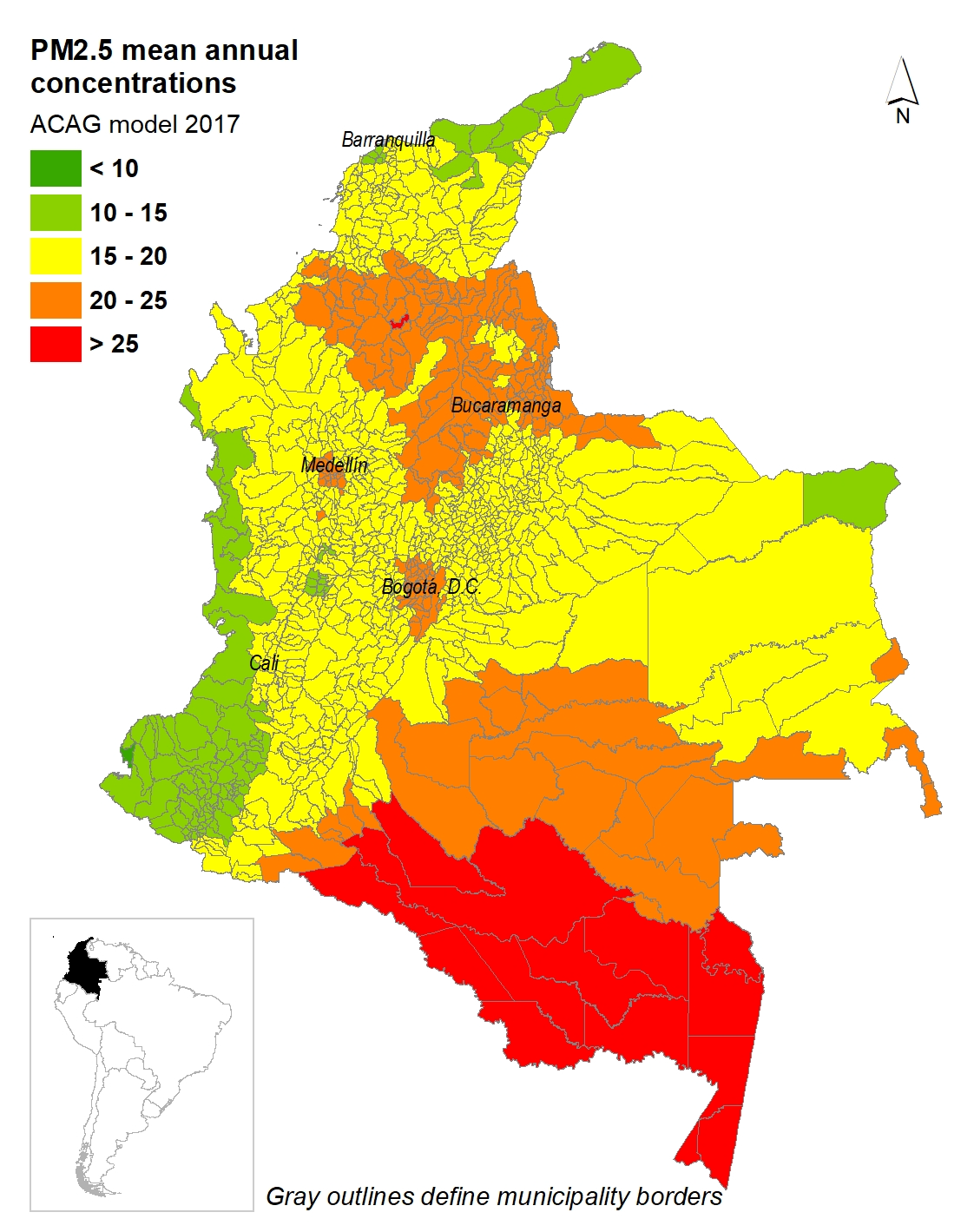 |
| 1. 2018   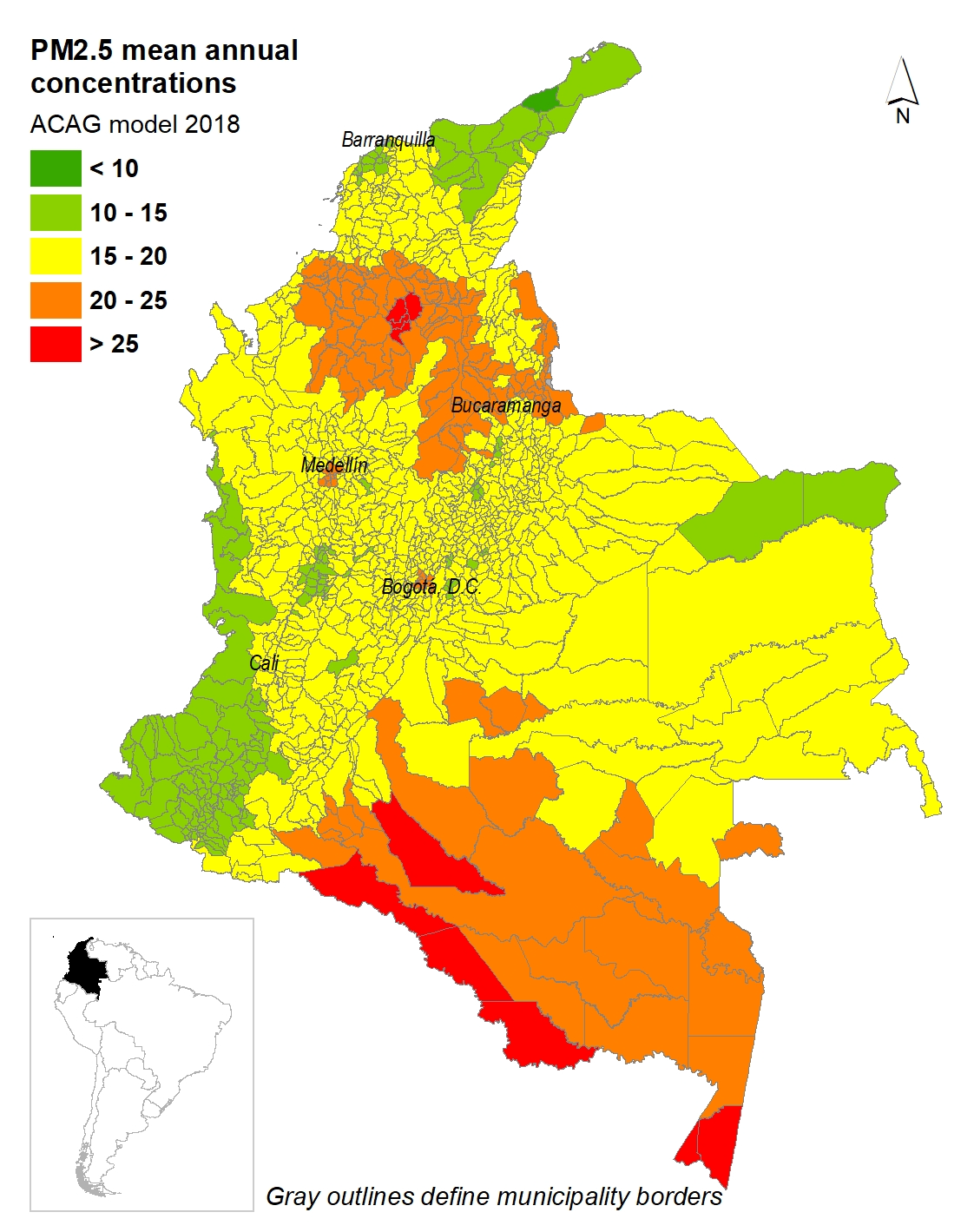 | 1. 2019   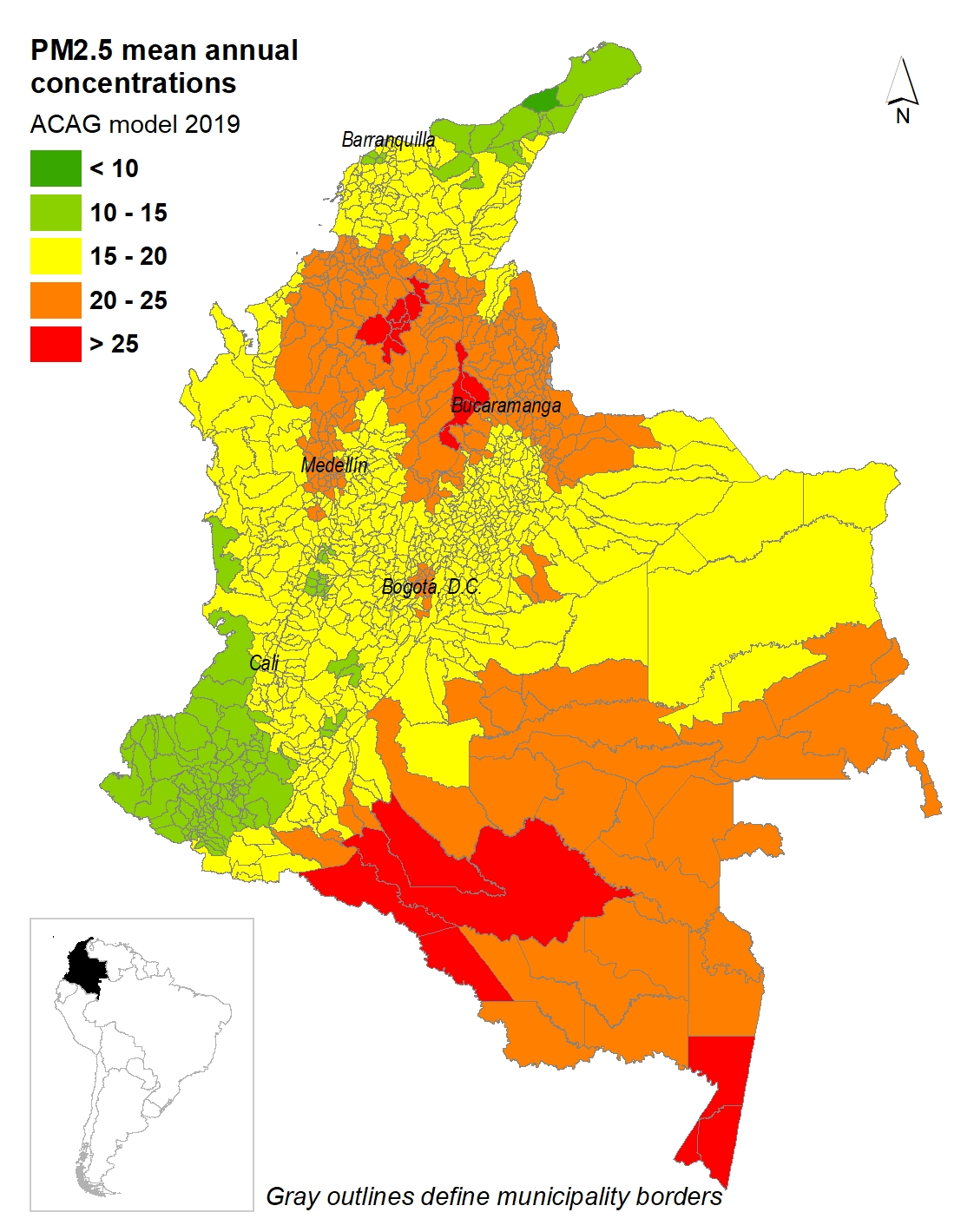 |

**Figure S2.** Estimations of surface PM_2.5_ mean annual concentrations at municipality level based on CAMSRA model, Colombia, 2014-2019

| 1. 2014   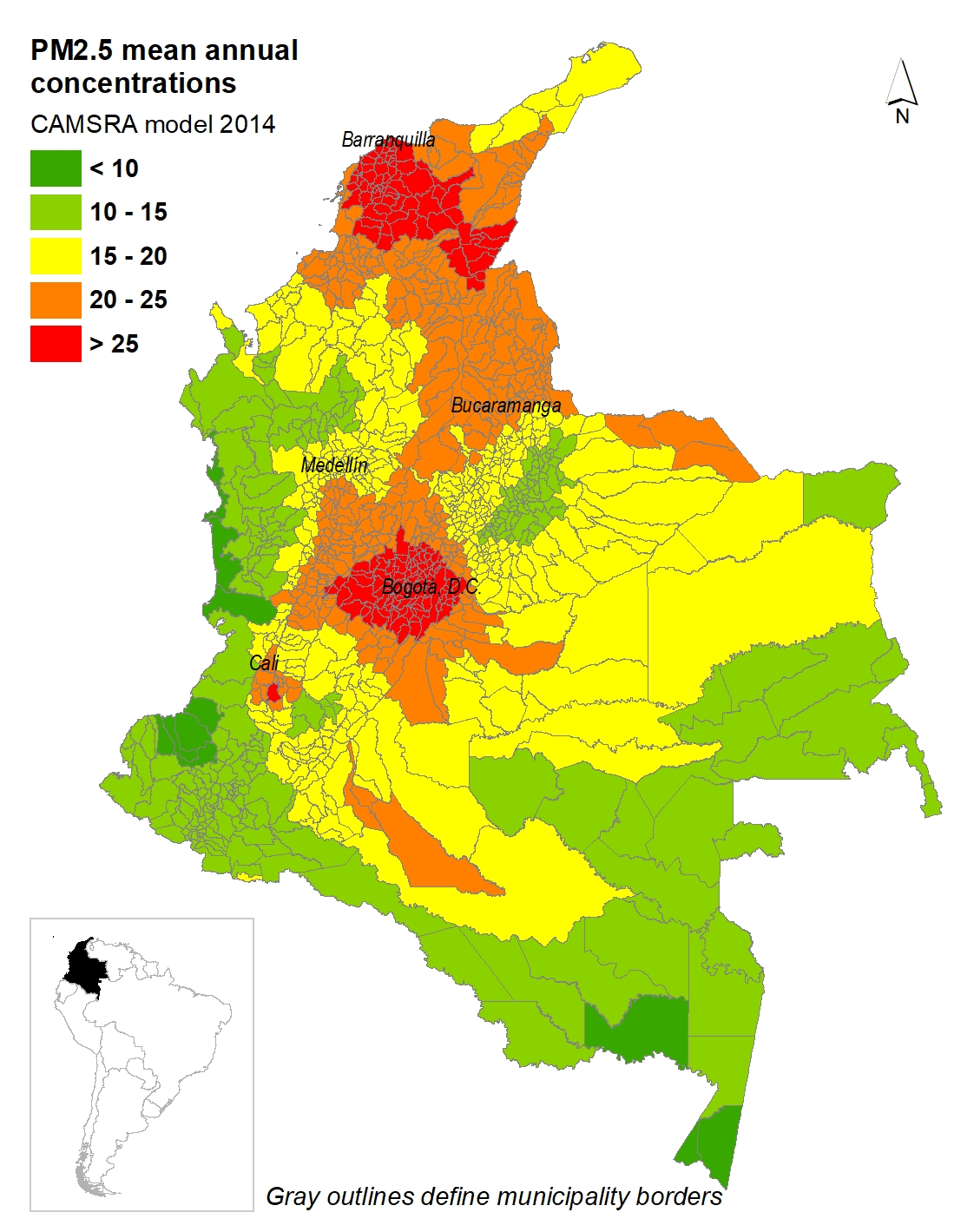 | 1. 2015   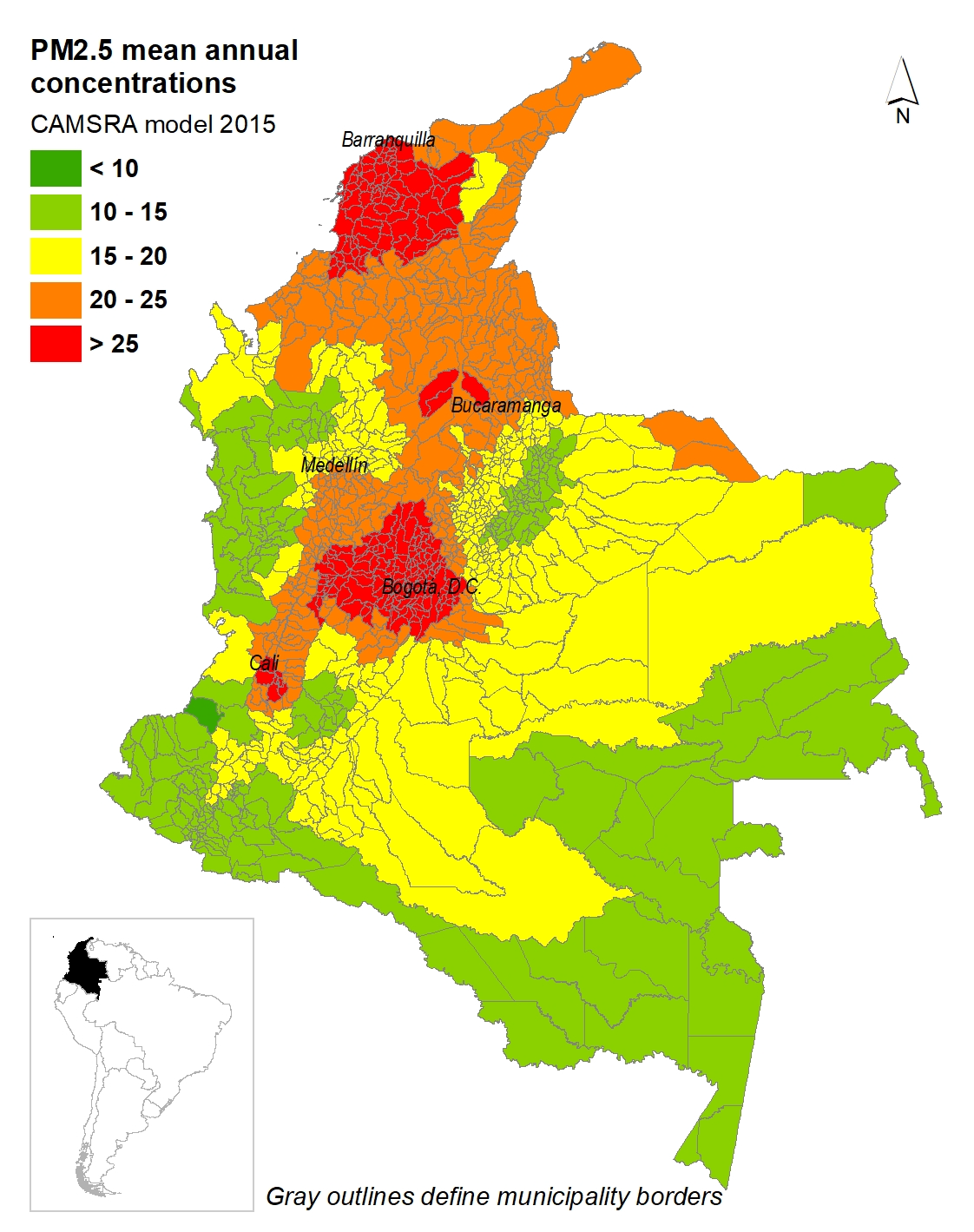 |
| --- | --- |
| 1. 2016   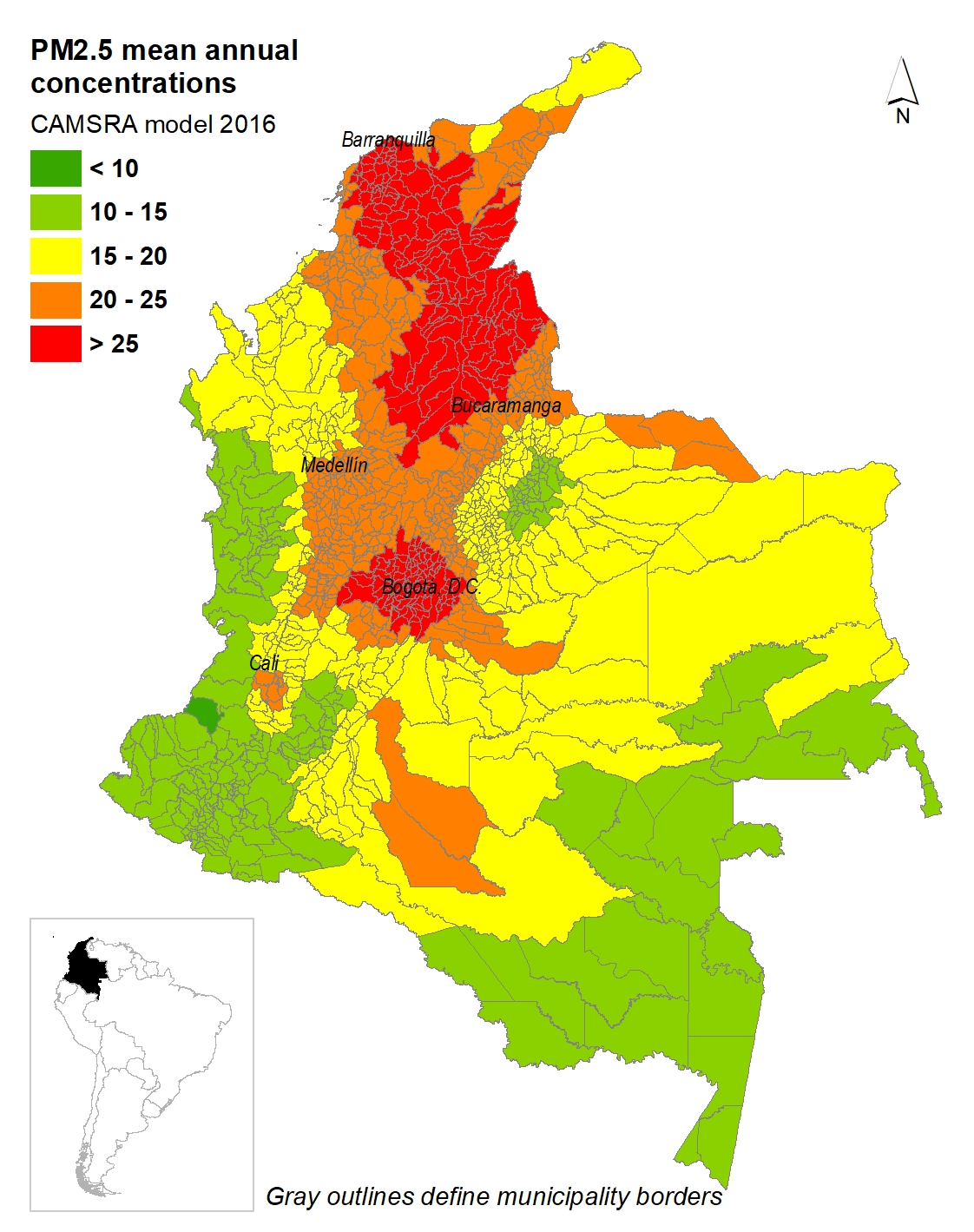 | 1. 2017   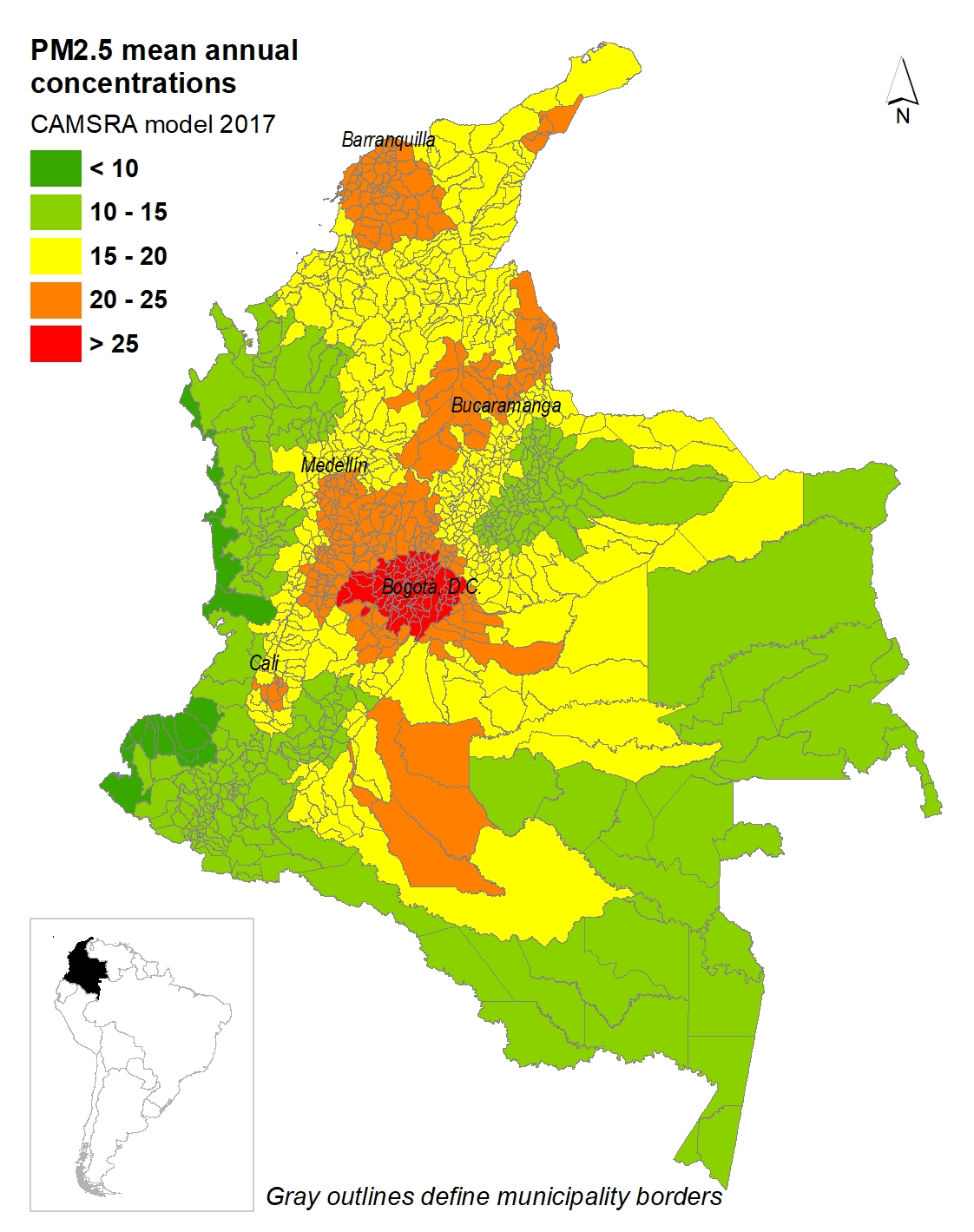 |
| 1. 2018   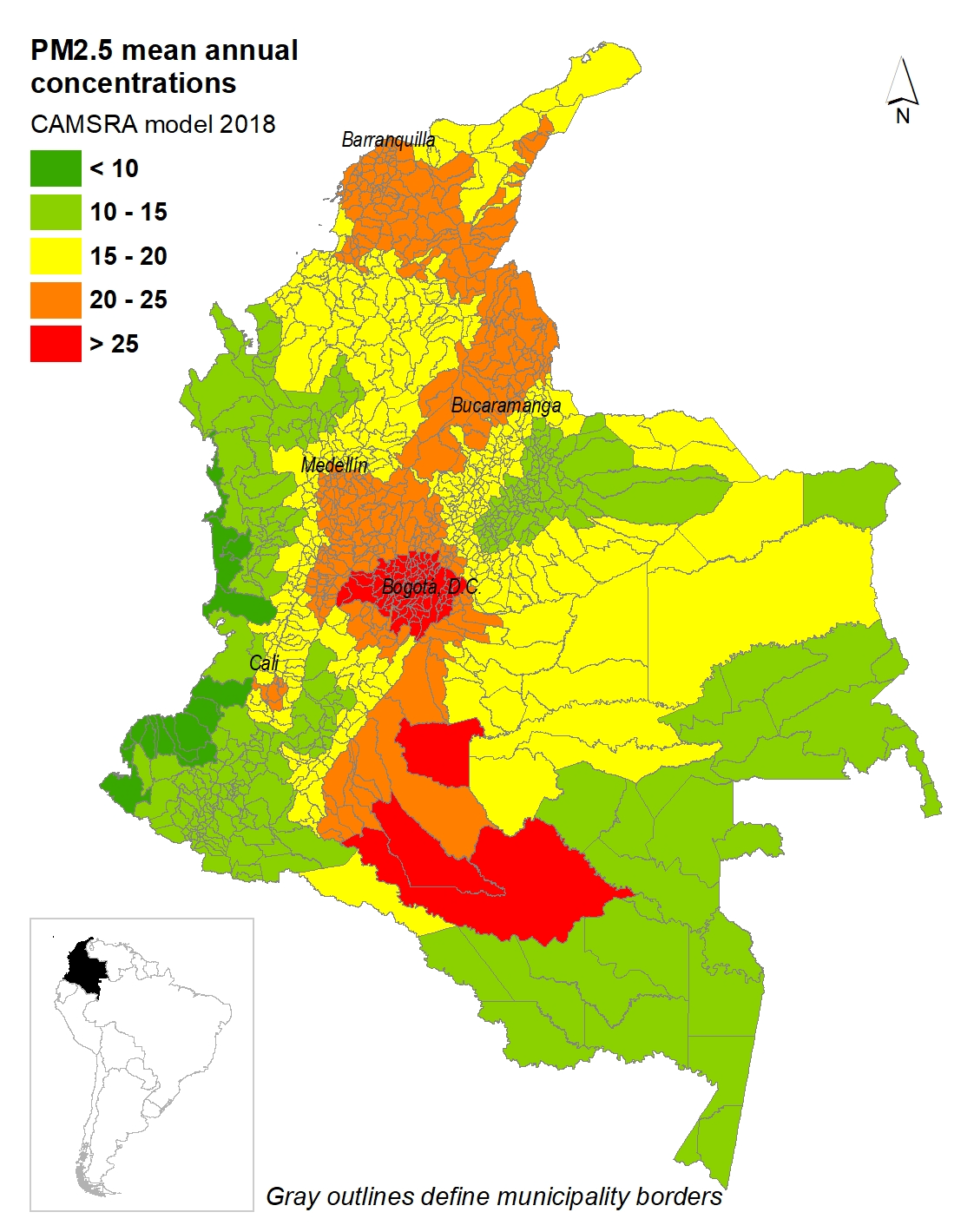 | 1. 2019   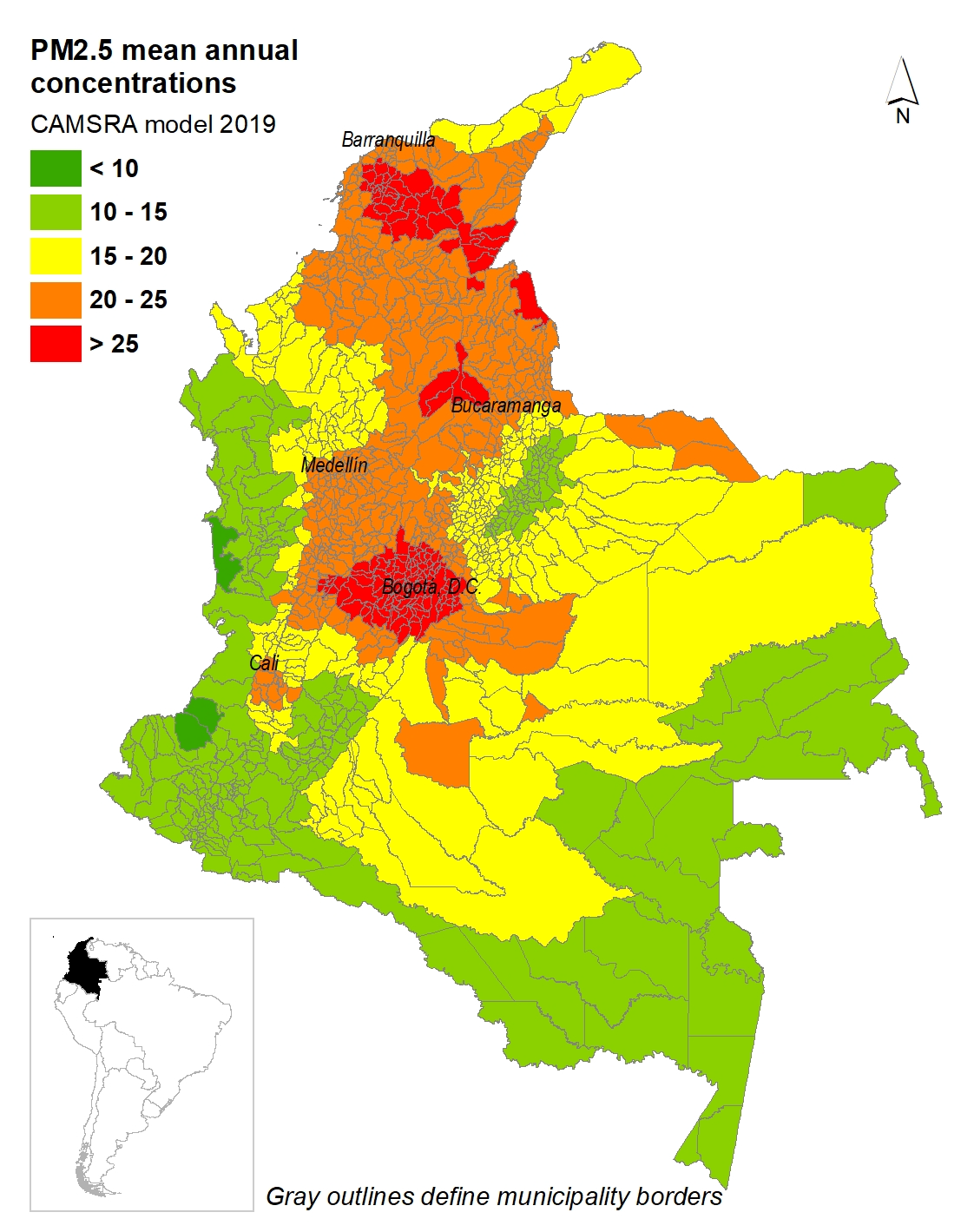 |
